# Emergence of Neonatal Sepsis Caused by MCR-9- and NDM-1-Co-Producing *Enterobacter hormaechei* in China

**DOI:** 10.3389/fcimb.2022.879409

**Published:** 2022-05-06

**Authors:** Chunlei Chen, Hao Xu, Ruishan Liu, Xinjun Hu, Jianfeng Han, Lingjiao Wu, Hao Fu, Beiwen Zheng, Yonghong Xiao

**Affiliations:** ^1^ Collaborative Innovation Center for Diagnosis and Treatment of Infectious Diseases, State Key Laboratory for Diagnosis and Treatment of Infectious Diseases, the First Affiliated Hospital, College of Medicine, Zhejiang University, Hangzhou, China; ^2^ Jinan Microecological Biomedicine Shandong Laboratory, Jinan, China; ^3^ Department of Laboratory Medicine, The First Affiliated Hospital of Zhengzhou University, Zhengzhou, China; ^4^ Department of Infectious Diseases, The First Affiliated Hospital, College of Clinical Medicine, Henan University of Science and Technology, Luoyang, China; ^5^ Sansure Biotech Inc. Medical Affairs Department, National Joint Local Engineering Research Center for Genetic Diagnosis of Infection Diseases and Tumors, Beijing, China; ^6^ Research Units of Infectious Diseases and Microecology, Chinese Academy of Medical Sciences, Beijing, China

**Keywords:** MCR-9, *Enterobacter cloacae* complex, neonatal, sepsis, IncHI2

## Abstract

Mobile colistin resistance (*mcr*) genes represent an emerging threat to public health. Reports on the prevalence, antimicrobial profiles, and clonality of MCR-9-producing *Enterobacter cloacae* complex (ECC) isolates on a national scale in China are limited. We screened 3,373 samples from humans, animals, and the environment and identified eleven MCR-9-positive ECC isolates. We further investigated their susceptibility, epidemiology, plasmid profiles, genetic features, and virulence potential. Ten strains were isolated from severe bloodstream infection cases, especially three of them were recovered from neonatal sepsis. *Enterobacter hormaechei* was the most predominant species among the MCR-9-producing ECC population. Moreover, the co-existence of MCR-9, CTX-M, and SHV-12 encoding genes in MCR-9-positive isolates was globally observed. Notably, *mcr-9* was mainly carried by IncHI2 plasmids, and we found a novel ~187 kb IncFII plasmid harboring *mcr-9*, with low similarity with known plasmids. In summary, our study presented genomic insights into genetic characteristics of MCR-9-producing ECC isolates retrieved from human, animal, and environment samples with one health perspective. This study is the first to reveal NDM-1- and MCR-9-co-producing ECC from neonatal sepsis in China. Our data highlights the risk for the hidden spread of the *mcr-9* colistin resistance gene.

## Introduction

*Enterobacter cloacae* complex (ECC) are clinically significant Gram-negative, facultatively anaerobic, rod-shaped pathogens, which cause severe nosocomial infections, including bloodstream infections, pneumonia, urinary tract infections, endocarditis, and intra-abdominal infections (Mezzatesta et al., 2012; Davin-Regli and Pages, 2015). It is worthy to note that ECC has now become the third broad spectrum Enterobacteriaceae species involved in hospital-acquired infections after *Escherichia coli* and *Klebsiella pneumoniae* (Potron et al., 2013). Currently, *E. cloacae* is the most frequent cluster of ECC isolated from adult intensive care unit (ICU) patients (Fernandez et al., 2015; Stoesser et al., 2015; Ferry et al., 2020), even in neonatal patients (Yaffee et al., 2016; Rahal et al., 2021).

*E. cloacae* complex produced an AmpC β-lactamase, which has intrinsic resistance to many front-line treatment options, including penicillins, penicillin-penicillinase inhibitor combinations, first-generation cephalosporins, and cefoxitin (Davin-Regli and Pages, 2015). It exhibits a high frequency of enzymatic resistance to third-generation cephalosporins when AmpC β-lactamases become overproduced (Ellington et al., 2019). Colistin has been the last resort of CRE treatment for several decades, which ensures it serves as an essential global priority for use to manage infections resistant to carbapenems and other β-lactams. Previous work evidenced that colistin show effective *in vitro* activities against severe infections caused by carbapenem-resistant ECC (Yang et al., 2018).

Worryingly, since the first mobile colistin resistance gene (*mcr-1*), conferring transmissible colistin resistance, was detected in 2015, ten major classes of *mcr* genes have been detected so far (Wang et al., 2020; Xu et al., 2021). Despite these reports, most of *mcr* genes prevalence in ECC has remained relatively lower than that in *E. coli* and *K. pneumoniae*, except for *mcr-9* (Macesic et al., 2021a). Since MCR-9 was identified in *Salmonella enterica* serotype Typhimurium in 2019 (Carroll et al., 2019), it was rapidly detected in several Enterobacteriaceae species worldwide (Osei Sekyere et al., 2020; Macesic et al., 2021a). Interestingly, few clinical isolates carrying the *mcr-9* gene exhibit colistin resistance phenotype, which may be due to the low-level gene expression (Macesic et al., 2021a). Of note, increasing reports of *E. cloacae* isolates co-harboring MCR-9, carbapenemases, and extended-spectrum β-lactamase genes (ESBLs) are being reported worldwide due to the dissemination of self-transmissible IncHI2 plasmids (Haenni et al., 2020; Umeda et al., 2021). A recent study identified an *mcr-9*/*bla*_NDM-1_-co-harboring plasmid of *E. cloacae* from a lethal adult bloodstream infection (BSI) case (Lin et al., 2020). Severe invasive infections caused by such isolates definitely limit treatment options for clinicians and threaten public health. However, MCR-9-producing ECC infections in neonatal patients have never been reported.

In this study, we did a retrospective study to screen and examine the virulence profile and genetic context of the *mcr-9*-carrying ECC isolates from human, animal, and environmental samples in China from 2010 to 2020. The epidemiology, genetic features, antimicrobial profiles, virulence potential, and global phylogenomic analysis of the isolates were further investigated. In general, we describe for the first time the emergence of the *mcr-9* colistin resistance gene in neonatal sepsis in China.

## Materials and Methods

### Study Design, Setting, and Bacterial Isolates

In this study, we screened MCR-9-producing ECC isolates recovered from human stool samples collected between 2010 and 2020, animal stool samples collected between 2016 and 2020, and bloodstream infection isolates from the Consortium of Blood Bacterial Resistant Investigation Collaborative Systems (BRICS) (Zheng et al., 2017b; Zheng et al., 2019a) collected between 2014 and 2021. ECC intestinal colonization isolates were collected from inpatients submitting specimens to the First Affiliated Hospital of Zhejiang University (Hangzhou, Zhejiang Province) and the First Affiliated Hospital of Zhengzhou University (Zhengzhou, Henan Province). Animal stool samples were retrieved from our previous studies (Zheng et al., 2017a; Feng et al., 2019; Zheng et al., 2019b; Zheng et al., 2019c). As a routine screening for ECC strains, the collected stool samples were plated on MacConkey agar (OXOID, Hampshire, UK) for 18-24h at 37°C. Preliminary bacterial identification was conducted by using MALDI-TOF MS (Bruker Daltonik GmbH, Bremen, Germany), and the identification of ECC species was confirmed by whole-genome sequencing-based genomic analysis. The *mcr-9* gene was detected by using PCR and sequencing (Macesic et al., 2021a). Clinical data were extracted from medical record systems. Ethical approval was granted by the Ethics Committee of the First Affiliated Hospital of Zhejiang University. Individual consent was obtained for all patients.

### Antimicrobial Susceptibility Testing

The minimum inhibitory concentrations (MICs) of different antimicrobial agents were determined by the microdilution method in Mueller-Hinton broth for all isolates. The tested antibiotics were aminoglycosides (gentamicin and amikacin), β-lactams (amoxicillin/clavulanic acid, aztreonam, cefotaxime, ceftazidime, cefpirome, imipenem, meropenem, and piperacillin/tazobactam), fluoroquinolones (levofloxacin and ciprofloxacin), tetracyclines (tetracycline and tigecycline), colistin, chloramphenicol, florfenicol, fosfomycin, and trimethoprim/sulfamethoxazole. AST results were interpreted according to the standards of the Clinical Laboratory Standards Institute (Clinical and Laboratory Standards Institute (CLSI), 2021). Breakpoints of colistin and tigecycline were interpreted following guidelines of the European Committee on Antimicrobial Susceptibility Testing (The European Committee on Antimicrobial Susceptibility Testing, 2020) (http://www.eucast.org/).

### Whole-Genome Sequencing

Total genomic DNA of ECC isolates was extracted using Gentra Puregene Yeast/Bact. Kit (Qiagen, Germany) according to the manufacturer’s protocol. Purified DNA was subjected to WGS on Illumina Novaseq (Illumina, Inc., CA, USA) system with the 150-bp paired-end approach and 150 × coverage. To further characterize the genetic environment of the *mcr-9* genes, we performed long-read sequencing using Nanopore sequencing technology for five isolates according to the different sizes of *mcr-9*-harboring plasmids. MinION (Oxford Nanopore Technologies, Oxford, UK) sequencing was prepared for isolates 51118, 52744, 53287, 60403, and 61363. DNA Libraries were constructed by using Ligation Sequencing Kits 1D (SQK-LSK109) to generate long contigs.

### *In Silico* MLST Analysis and Identification of Antimicrobial Resistance Genes and Plasmids

Raw reads were assembled into a number of scaffolds using Velvet 1.1 (Zerbino and Birney, 2008). Annotation was carried out by using Prokka (Seemann, 2014). Bacterial identification was conducted by average nucleotide identity (ANI) analysis with JSpeciesWS (Richter et al., 2016). Multi-locus sequence typing (MLST) analysis was performed as described previously (Cerqueira et al., 2017). The detection of antimicrobial resistance genes (ARGs) were identified using the ResFinder 2.1 database (Zankari et al., 2012) based on the identities (%). Plasmid replicons were identified using PlasmidFinder 2.0 (Carattoli and Hasman, 2020). Average nucleotide identity blast (ANIb) analysis was completed using pyani with default settings (https://github.com/widdowquinn/pyani). ANIb data for each strain were visualized by heatmaps.

### Phylogenetic Analysis

To further characterize the phylogenetic structure of MCR-9-producing ECC isolates, our collection of MCR-9-carrying isolates was supplemented with strains from the global collection. Genomes of 70 MCR-9-carrying ECC isolates were downloaded from the National Center for Biotechnology Information (NCBI) for comparative genomic analysis (**Table S1**). The phylogenies of the MCR-9-producing strains were performed by ROARY software based on the core genome SNPs (Sitto and Battistuzzi, 2020). Core genes were defined as described previously (Holt et al., 2015). Phylogenetic tree visualizations were produced using the Interactive Tree of Life (https://itol.embl.de/).

### Conjugation Assay

Conjugation experiments of all MCR-9-positive isolates were carried out using broth-based methods with *E. coli* J53 (azide-resistant) as the recipient strain. Transconjugants were selected on MH agar plates containing 150 μg/mL sodium azide plus 2 μg/mL colistin. The presence of *mcr-9* in transconjugants was confirmed by PCR and Sanger sequencing.

### Plasmid Analysis

Plasmid characterization was carried out by nuclease digestion pulsed-field gel electrophoresis (S1-PFGE), and Southern blot to estimate the size of the *mcr-9*-carrying plasmids (Zheng et al., 2016). The sequence of the target plasmids was assembled using plasmidSPAdes (Antipov et al., 2016). The sequences of representative plasmids were compared against other NCBI accessioned plasmid sequences using BLAST and plotted by BLAST Ring Image Generator (BRIG) (http://brig.sourceforge.net/). To identify targeted plasmids, a BLAST similarity search against the GenBank NT database was performed for each scaffold. Plasmid sequences were defined when > 70% of the scaffold length matched the plasmid sequences and < 30% compared either the mobile element or chromosomal sequences based on MUMMER alignment (Delcher et al., 2003). The best BLAST hit to each scaffold was manually inspected with the PLSDB database, a resource of complete bacterial plasmids (Galata et al., 2018).

### Serum Killing Assay and *In Vivo Galleria mellonella* Infection Model

The virulence of MCR-9-producing ECC strains was tested in the serum killing assay and *G. mellonella* model of infection. Serum killing assay was performed as described previously (Shen et al., 2020). Briefly, fresh nonheated human serum (NHS) from different healthy individuals and frozen the samples at -80°C. The serum bactericidal effect was presented as survival curves and rates, using the following formula: Bacterial survival rate = (number of colonies with normal serum/number of colonies with inactivated serum) × 100%. Each experiment was performed in triplicate.

For *G. mellonella in vivo* infection assay, every 10 *G. mellonella* weighing about 250 mg were randomly divided into groups, and each *G. mellonella* was injected with 20ul 1 × 10^7^ CFU/mL concentration of bacteria. In addition, the negative control group was injected with only 20ul PBS. All *G. mellonella* were cultured at 37°C, and the status of *G. mellonella* was observed every 12h for seven consecutive days (Niu et al., 2020). *E. coli* ATCC 25922 was used as the quality control strain.

## Statistical Analysis

The R studio software was used for statistical analysis. A P-value of 0.05 was used as the significance level; P<0.05 indicated statistically significant differences.

## Results

### Bacterial Isolation and Identification

**Figure 1:**
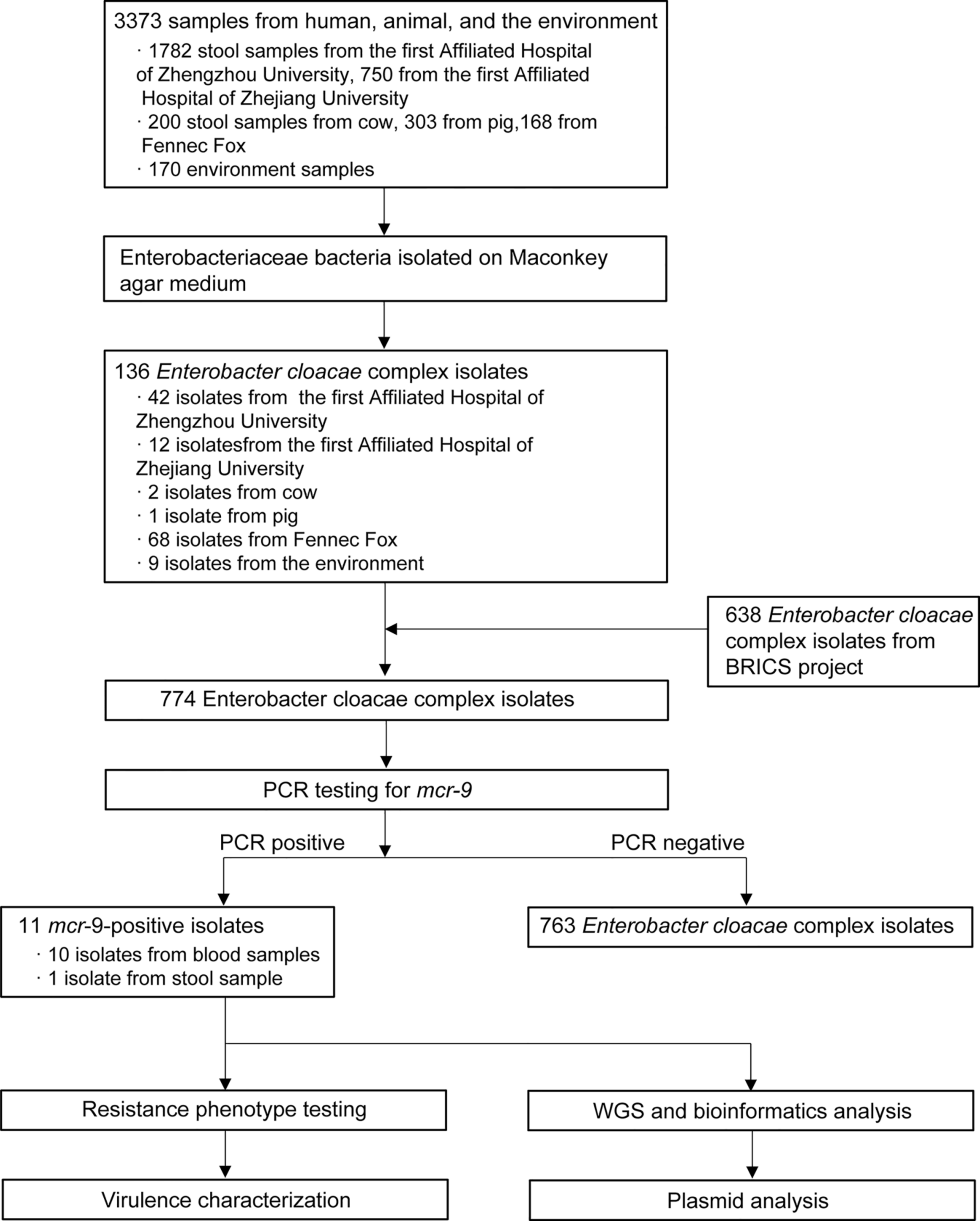
Schematic procedure of the study. BRICS, Blood Bacterial Resistant Investigation Collaborative Systems; WGS, whole-genome sequencing.

In general, we screened 1,782 stool samples from the First Affiliated Hospital of Zhengzhou University, 750 from the First Affiliated Hospital of Zhejiang University, 200 stool samples from cow, 303 from pig, 168 from fennec fox, and 170 from environment samples (**Figure 1**). Of the 3,373 samples from humans, animals, and the environment, a total of 136 ECC isolates were obtained in this study. Of 136 isolates, 56 recovered from stool sample, 71 from animals, and 9 from environment. Combined with 638 strains collected from the BRICS project, 774 ECC isolates were screened for MCR-9. As a result, eleven MCR-9 producing ECC strains (1.42%, 11/774) from 2010 to 2017 were identified in this work. Ten MCR-9-postive isolates were recovered from blood sample and one from stool sample.

### Emergence of MCR-9-Producing ECC in Neonatal Sepsis

**Figure 2:**
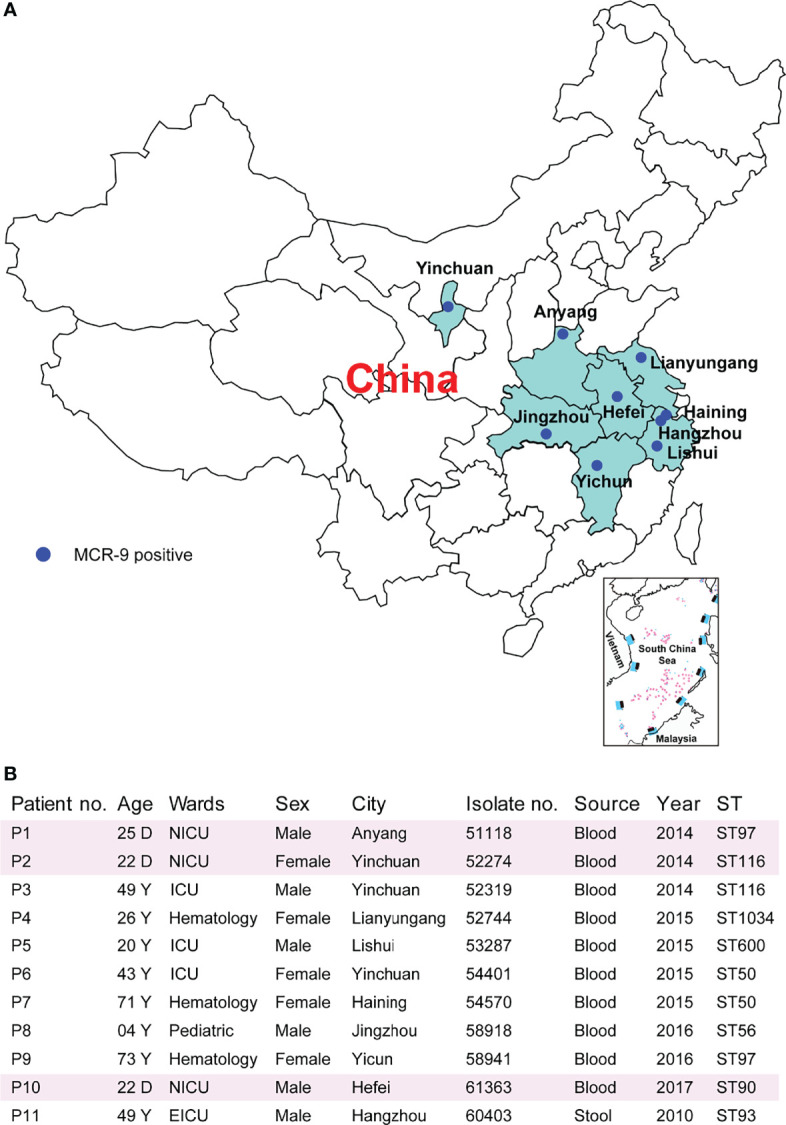
Characteristics of MCR-9-producing ECC-associated cases. **(A)** Study sites and geographical distribution of MCR-9-producing ECC isolates. Seven provinces shown in color were included in the surveillance for MCR-9. **(B)** Clinical characteristics and epidemiological characteristics of MCR-9-producing ECC-associated isolates.

Characteristics of the 11 ECC-associated cases are summarized in **Figure 2**. Of these cases, patients came from nine cities located in seven provinces (**Figure 2A**). It is worthy to note that three isolates were recovered from neonatal patients, one from child and seven from adults (**Figure 2B**). The isolates originated from patients with primary bloodstream infections (n = 10) and colonization (n = 1). The eleven isolates were all identified by MALDI-TOF MS as *E. cloacae.* The ANIb analysis revealed that nine isolates were *Enterobacter hormaechei*, and the other two were *Enterobacter kobei* (52744 and 58918) (**Figure S1**). Of note, two *E. hormaechei* isolates and one *E. kobei* were identified from neonatal sepsis cases.

### Phenotypic and Molecular Characteristics of Eleven Isolates

Resistance determinants, plasmid replicons and STs are outlined in **Table S1**. The isolates were found to belong to ST50 (n = 2), ST97 (n = 2), ST116 (n = 2), ST56 (n = 1), ST90 (n = 1), ST93 (n = 1), ST600 (n = 1), and ST1034 (n = 1) (**Figure 2B**). Isolates were resistant to multiple antimicrobial classes, including amoxicillin/clavulanic acid (100%), cefotaxime (100%), ceftazidime (100%), and cefpirome (91%), but remained susceptible to amikacin (91%), tigecycline (91%) and fosfomycin (82%) (**Table S2**). Despite the presence of *mcr-9*, nine *E. hormaechei* isolates were colistin susceptible (MICs ≤ 2 mg/). In contrast, two *E. kobei* exhibit high level colistin resistance (MICs ≥ 64 mg/)

### *E. hormaechei* Is Predominant in the MCR-9-Producing ECC Population

**Figure 3:**
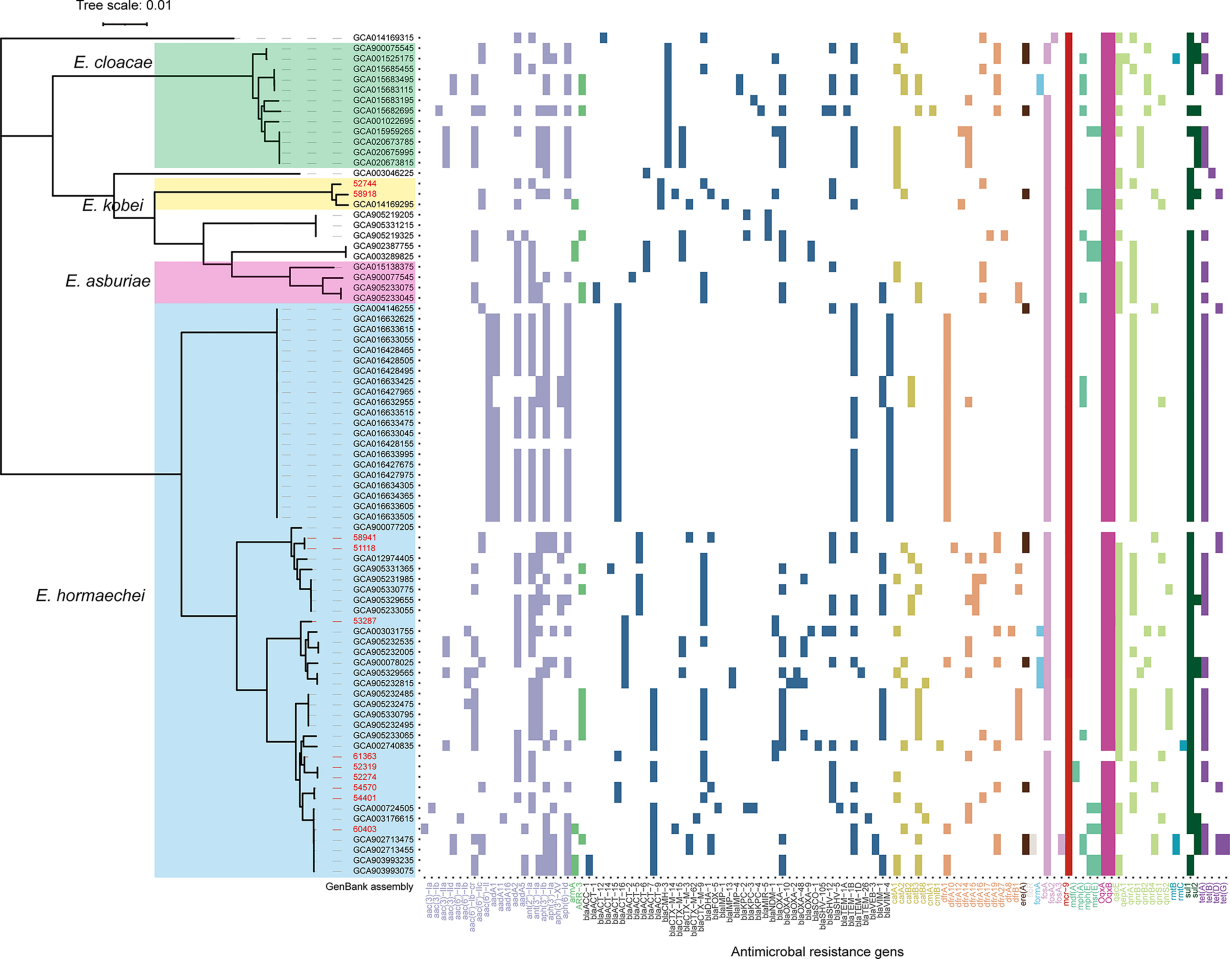
Core-genome phylogeny and resistome of 70 MCR-9-positive ECC isolates from NCBI genome database and the eleven isolates from this work. *E. hormaechei*, *E. cloacae*, *E. kobei*, and *E. arburiae* isolates are indicated in blue, green, yellow, and pink. Scale bars represent the number of substitutions per site. The ARGs distribution of MCR-9-positive ECC isolates is presented on the right. The ARGs groups are indicated with different colors. Isolates identified in this study are shown in red.

Core-genome phylogenetic analysis classified the 81 MCR-9-positive isolates into four clades (**Figure 3**). The 55 (57.9%) MCR-9-producing *E. hormaechei* strains constitute the largest clade, followed by *E. cloaece* (n = 12). Of note, two ST116 *E. hormaechei* isolates (52274 and 52319) recovered from the same hospital were phylogenetically closely related, indicating clonal dissemination in the region. Surprisingly, seven ST93 *E. hormaechei* isolates were identified from China, the USA, France, and Spain, suggesting global transmission.

### Co-Existence of MCR-9, CTX-M, and SHV-12 Encoding Genes Globally

ESBL genes were detected in all isolates identified in this work (**Figure 3**). Nine of the eleven isolates were positive for *bla*_CTX-M_, with *bla*_CTX-M-9_ the most prevalent (n = 6), followed by *bla*_CTX-M-14_ (n = 2) and *bla*_CTX-M-3_ (n = 1). Three isolates (58918, 53287, and 61363) were found to carry *bla*_NDM-1_ gene. WGS determined that *bla*_NDM-1_ was localized on a ~45 kb IncX3 plasmid and a ~60 kb IncN plasmid in isolates 53287 and 61363, respectively (**Figure S2**). The *bla*_SHV-12_ gene was detected in eight of the eleven ESBL-positive isolates. AmpC β-lactamase gene, *bla*_ATC_, was detected in all ESBL-positive isolates. *In silico* analysis found that CTX-M-9/SHV-12/MCR-9 positive ECC isolates are prevalent in a global collection, which indicated that these three genes probably co-localized on a plasmid.

### *MCR-9* Was Mainly Carried by IncHI2 Plasmids

**Figure 4:**
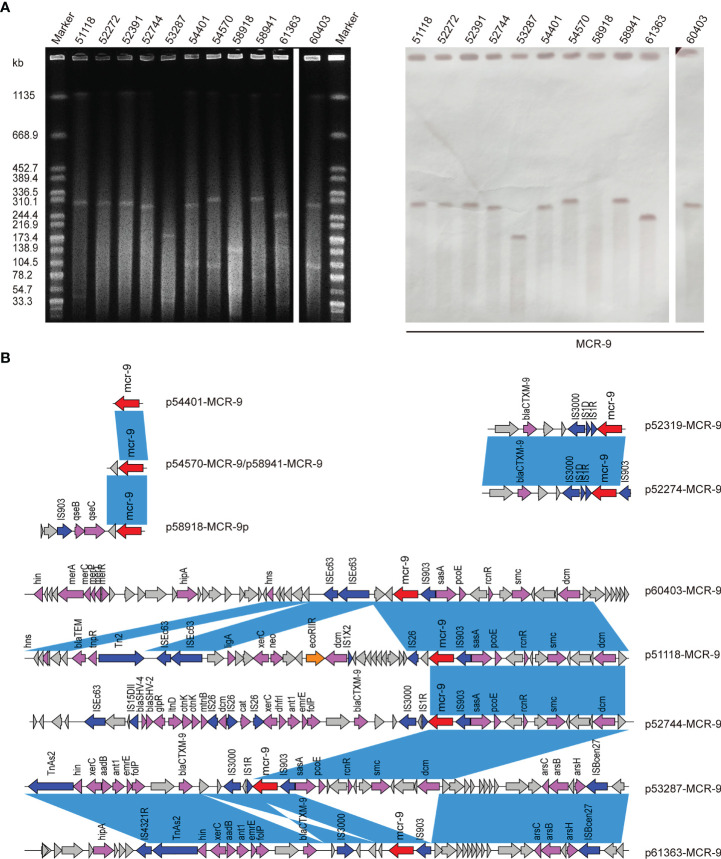
**(A)** S1-PFGE and Southern blot of eleven MCR-positive isolates. S1-PFGE patterns of Salmonella isolates and their relevant conjugants. Southern blot-hybridization of *S1*-nuclease digested DNA using a specific probe (*mcr-9*). M: *Xba*I digested total DNA of *Salmonella enterica* serotype Braenderup H9812 as a size marker. **(B)** Genetic contexts of *mcr-*9 genes in eleven ECC isolates. Blue rectangles highlight identical regions.

**Figure 5:**
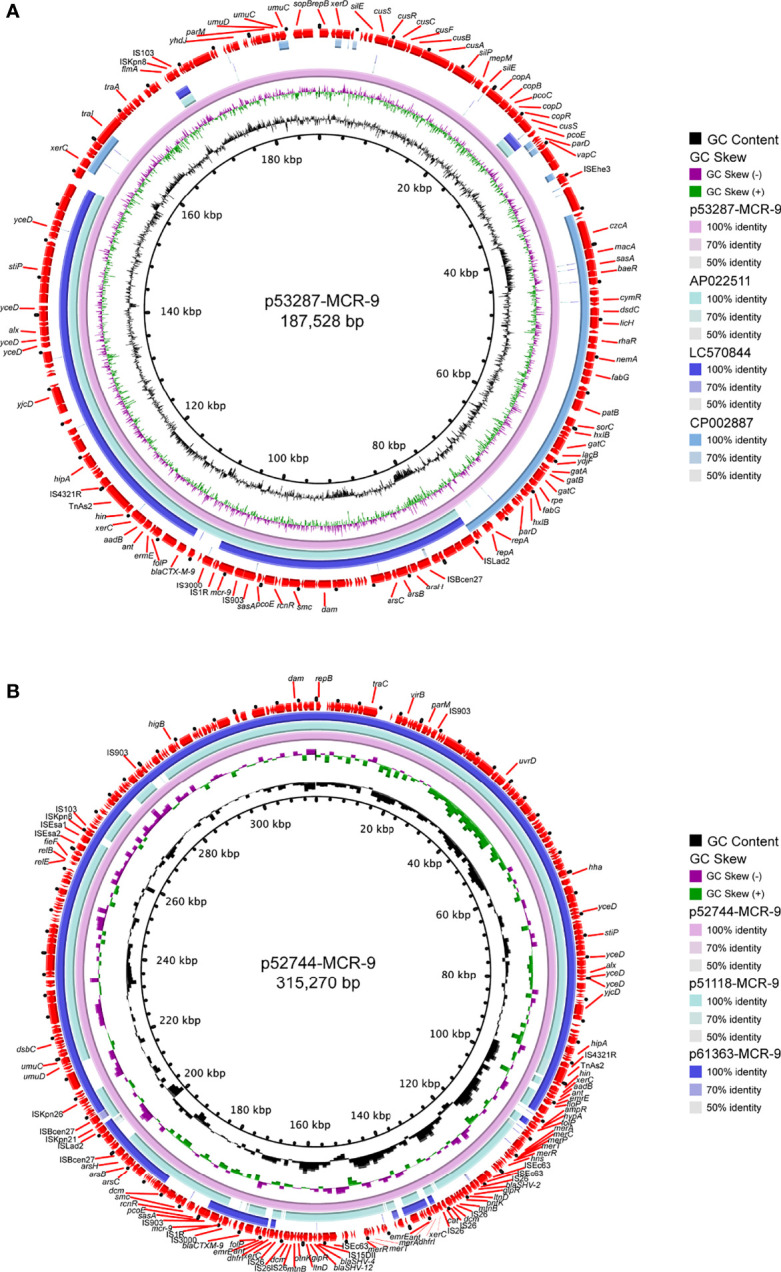
Circular representation of the studied *mcr-9*-carrying plasmids. **(A)** Analysis of the IncFII plasmid p53287-MCR-9 carried by the bacteremia associated *E hormaechei* strain 53287. **(B)** Sequence comparison of the *mcr-9*-encoding IncHI2 plasmids p57244-MCR-9, p51118-MCR-9, and p61363-MCR-9. GC content and GC Skew were represented on the inner map’s distance scale (kb). Each plasmid was compared to its most closely-related plasmid. The red arc around the map indicated ORFs. Certain important genes were also indicated on the ring.

S1-PFGE and Southern blot revealed that *mcr-9* was carried by plasmids in ten isolates, except isolate 58918, which is chromosome encoded (**Figure 4A**). We further identified ten contigs carrying *mcr-9* gene from genome sequences. All were typed as IncHI2 plasmid ST1, except for p53287-MCR-9, which was classified as IncFII (**Table S1**). IncHI2 plasmid was ~244-330 kb in size and carried many mercury, tellurium, copper, and antimicrobial resistance genes (**Figures 4**, **5**). These ARGs included resistance determinants for aminoglycosides, β-lactams, fluoroquinolones, macrolides, sulphonamides, tetracyclines, and trimethoprim. Conjugation experiments confirmed eight MCR-9-carrying plasmids were self-transmissible at 37 or 26°C (**Table S1**). In contrast, transconjugants containing p53287-MCR-9 and p60403-MCR-9 were not obtained, despite the filter-mating experiments were repeated at 25 to 37°C, suggesting that these plasmids could not be transferred. Examining the complete sequence of p53287-MCR-9 revealed that there were incomplete conjugative transfer loci on this plasmid (**Figure 5A**).

### Genetic Environment of *MCR-9* Genes

The genetic environment of *mcr-9* regions and plasmid backbone differed in our isolate collection, suggesting a diversity of MCR-9-carrying plasmids (**Figure 4B**). In p58918-MCR-9, two genes proposed to regulate the expression of colistin resistance, *qseC* and *qseB*, are localized downstream of *mcr-9*.1 and upstream of IS*903*. p52319-MCR-9 and p52274-MCR-9 shared a similar genetic context of *mcr-9.* Of note, the conserved region in *mcr-9*-harboring plasmids encoded the nickel-cobalt efflux transporters (*rcnR*/*rcnS*) and sensory protein kinases (*pcoS*/*pcoE*) (**Figure 4B**). However, the downstream region of *mcr-9* was highly varied between plasmids (**Figure 5B**).

### Virulence of MCR9-Producing ECC Isolates

**Figure 6:**
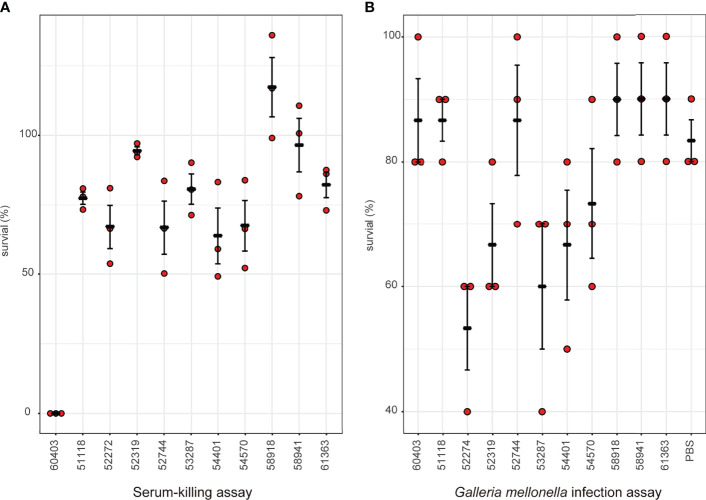
Virulence profiles of MCR-9-positive ECC isolates. **(A)** Serum-killing assay. Survival in a serum-killing assay of ECC strains. The survival is denoted in percentage. The bars denote means and standard errors of the mean. **(B)** *Galleria mellonella* infection assay. Survival at 144 h in a *G mellonella* assay of ECC strains. The survival is denoted in percentage. The bars denote means and standard errors of the mean.

Considering the emergence of MCR-9-producing ECC in neonatal sepsis cases, we further assessed the virulence profiles of these isolates. The serum survivability was >95% for three isolates and ranged from 65 to 83% for seven isolates (**Figure 6A**). In contrast, isolate 60403 showed low serum survivability. The *G. mellonella* infection assay revealed that seven isolates observed high survivability (> 84%) infection, while the other five isolates caused intermediate survivability (53–73%) (**Figure 6B**).

## Discussion

Thus far, the ECC species remain largely susceptible to several classes of antimicrobial agents used in China (Liu et al., 2015; Yang et al., 2018; Hu et al., 2020). However, the rapid increase of nosocomial infections caused by carbapenem-resistant ECC has raised growing worrisome worldwide, which result in the reuse of colistin to treat bacterial infections (Wang et al., 2018). Plasmid-mediated *mcr* colistin resistance genes can be transferred across Enterobacteriaceae species and result in global dissemination, representing a significant threat to public health (Zheng et al., 2018a; Zheng et al., 2018b). The putative colistin resistance gene *mcr-9* was first identified by WGS to confer phenotypic resistance to colistin in Enterobacteriaceae (Carroll et al., 2019). Screening of the *mcr-9* gene in isolates received from animals, the environment, and humans is necessary to understand its global or national spread. Before the current study, the prevalence, antimicrobial profiles, and clonality of MCR-9-producing ECC on a national scale in China were unclear. We screened our biobank over 3,000 various samples and identified *mcr-9* in eleven isolates. Our study reveals widespread this newly identified colistin resistance gene, *mcr-9*, among bloodstream infection isolates.

We report here a relatively low prevalence of MCR-9 in ECC isolates, with a positivity rate of 1.42% (11/774), although it was higher than the 0.18% (60/33,205) rate in *Salmonella enterica* reported by Yanan Wang *et al.* (Wang et al., 2021). Since it was first detected in the USA (Carroll et al., 2019), MCR-9 has been found in five continents from various human, animal, environmental, and food samples (Bitar et al., 2020; Kamathewatta et al., 2020; Osei Sekyere et al., 2020; Elbediwi et al., 2021; Ha et al., 2021; Khodor et al., 2021). Our findings highlight the unexpected spread of MCR-9 and emphasize the need for close surveillance of antimicrobial resistance in bloodstream infection isolates.

Remarkably, among our *mcr-9*-positive isolates, all of them encoded aminoglycoside resistance, fosfomycin resistance, sulfonamide resistance, and β-lactamase genes, raising the concern that the spread of *mcr-9* might also be related to the use of these antimicrobial agents in China. In this study, *bla*_CTX-M-9_ was co-existed with *mcr-9* in the same plasmid. Interestingly, *bla*_CTX-M-9_ was previously found as the most prevalent ESBL gene in nosocomially acquired ECC in China (Zhou et al., 2018). The discovery of *bla*_CTX-M-9-_ and *mcr-9-* co-harboring plasmid is of grave concern, as an epidemic could have potentially serious consequences.

Carbapenem-resistant Enterobacteriaceae (CRE) are important pathogens causing serious community-acquired and nosocomial infections globally (Zheng et al., 2019a; Zheng et al., 2020). Previously, MCR-9 has been identified in several CRE backgrounds since it was described in 2019 (Macesic et al., 2021b). Additionally, co-production of NDM-1 and MCR-9 in ECC isolates was mainly identified from China (Yuan et al., 2019; Faccone et al., 2020; Khalifa et al., 2020; Lin et al., 2020; Ai et al., 2021; Ding et al., 2021; Liu et al., 2021; Sun et al., 2021), these studies indicated that the extensive use of carbapenems in China might drive the occurrence of these multidrug-resistant ECC isolates. It is worthy to note that we first detected the NDM-1- and MCR-9-co-producing ECC isolates cause neonatal sepsis. Our data reveal the likelihood of wide dissemination of NDM-1 and MCR-9 in pediatric or neonatal intensive care units (PICU/NICU). This finding highlights the unexpected spread of NDM-1and MCR-9 and emphasizes the urgent need for effective surveillance of antimicrobial resistance in PICU/NICU. This linkage is a matter of concern since it could herald the possibility of a co-spread of the two genes, both involved in resistance to last-resort drugs (Lin et al., 2020).

IncHI2 plasmids were found to be the predominant replicon type carrying *mcr-9*, and the conserved *rcnR-rcnA-pcoE-pcoS*-IS*903*-*mcr-9-wbuC* structure exists in most *mcr-9* cassettes (Macesic et al., 2021b). It was widely detected in various Enterobacteriaceae species (Bitar et al., 2020; Borjesson et al., 2020; Campos-Madueno et al., 2021; Liu et al., 2021; Simoni et al., 2021; Umeda et al., 2021). The observation in this work is consistent with previous investigations, and nine *mcr-9* genes were carried by IncHI2 plasmid. Moreover, we identified a novel *mcr-9*-harboring IncFII plasmid in this work, which shared a relatively low similarity with any known plasmids. The spread of this plasmid in clinical settings should be closely monitored.

## Conclusion

This is the first investigation aimed to screen the MCR-9-producing ECC isolates from clinical, healthy human, animal, and environment samples with one health perspective. This work also first detected the MCR-9-producing ECC from neonatal sepsis in China. We further reported the complete sequence of plasmids harboring the *mcr-9* and *bla*_NDM-1_ genes. The *mcr-9* gene was mainly located on IncHI2 plasmids. Our data highlights the risk for the hidden spread of the *mcr-9* colistin resistance gene.

## Data Availability Statement

The datasets presented in this study can be found in online repositories. The names of the repository/repositories and accession number(s) can be found in the article/**Supplementary Material**.

## Ethics Statement

Ethical approval was granted by the Ethics Committee of the First Affiliated Hospital of Zhejiang University. Individual consent was obtained for all patients.

## Author Contributions

BZ and YX designed the study. BZ wrote the original manuscript. CC and HX collected the data and performed the data analyses. CC provided medication guidance. CC and RL involved in patient management and provided clinical information. HX, RL, XH, LW and HF performed laboratory tests. JH revised and supervised the paper. All authors contributed to the article and approved the final manuscript.

## Funding

We gratefully acknowledge the financial support of the National Natural Science Foundation of China (82072314 and 81971984), the Research Project of Jinan Microecological Biomedicine Shandong Laboratory (JNL-2022006B and JNL-2022011B), and CAMS Innovation Fund for Medical Sciences (2019-I2M-5-045).

## Conflict of Interest

Author JH was employed by Sansure Biotech Inc.

The remaining authors declare that the research was conducted in the absence of any commercial or financial relationships that could be construed as a potential conflict of interest.

## Publisher’s Note

All claims expressed in this article are solely those of the authors and do not necessarily represent those of their affiliated organizations, or those of the publisher, the editors and the reviewers. Any product that may be evaluated in this article, or claim that may be made by its manufacturer, is not guaranteed or endorsed by the publisher.

## Supplementary Material

Supplementary Figure 1 ANIb analysis of MCR-9-producing ECC isolates.

Supplementary Figure 2 Circular representation of the studied *bla*_NDM-1_-carrying plasmids. **(A)** Sequence comparison of the IncX3 plasmid p53287-NDM-1carried by the bacteremia associated with *E. hormaechei* strain 53287. **(B)** Sequence comparison of the *bla*_NDM-1_-carrying IncN plasmid p61363-NDM-1. GC content and GC Skew were represented on the inner map’s distance scale (kb). Each plasmid was compared to its most closely-related plasmid. The red arc around the map indicated ORFs. Certain important genes were also indicated on the ring.
