# Supplementary material for: Emergence of Neonatal Sepsis Caused by MCR-9- and NDM-1-Co-Producing Enterobacter hormaechei in China
Source: Front Cell Infect Microbiol. 2022 May 6;12:879409. doi: 10.3389/fcimb.2022.879409 (PMC9120612; doi:10.3389/fcimb.2022.879409)
Supplement: Supplementary Figure 1 — ANIb analysis of MCR-9-producing ECC isolates. [file DataSheet_1.pdf]

Table S1. The detailed information of genomes of 81 MCR-1-producing ECC isolates collected in this study and downloaded from public database for comparison of genetic features.

Strain	Species	Assembly no.	BioSample no.	Year	Country*	Host	ST	Plasmid replicon types
ECNIH2	*E. hormaechei*	GCA_000724505	SAMN02713682	2012	USA	Environment	93	IncHI2, IncHI2A, RepA_1_pKPC-CAV1321, IncA/C2, IncN
GN02616	*E. cloacae*	GCA_001022695	SAMN03732719	2007	USA	Human	608	IncHI2, IncHI2A, RepA_1_pKPC-CAV1321, Col440I, ColRNAI, Col440II
SMART_1493	*E. cloacae*	GCA_001525175	SAMN04431024	2014	Viet Nam	Human	462	IncHI2, IncHI2A, RepA_1_pKPC-CAV1321, IncFII, IncN, Col440I, ColRNAI, Col440II
20ES	*E. hormaechei*	GCA_002740835	SAMN07452577	2013	Romania	Human	254	IncHI2, IncHI2A, RepA_1_pKPC-CAV1321, IncL/M, IncFII, IncFIB, IncR, IncFIA, Col440I, ColRNAI, Col440II
174	*E. hormaechei*	GCA_003031755	SAMN04456586	2015	USA	Human	231	IncHI2, IncHI2A, RepA_1_pKPC-CAV1321, IncL/M, IncFIA, IncR
ENT1	*E. asburiae*	GCA_003046225	SAMN08848070	2016	Brazil	Environment	New	IncFIB, IncFII, IncHI2, IncHI2A, RepA_1_pKPC-CAV1321, Col440I, ColRNAI
TUM9991	*E. hormaechei*	GCA_003176615	SAMD00115713	2010	NA	Human	93	IncHI2, IncHI2A, RepA_1_pKPC-CAV1321, IncN, IncFII
99B3	*E. asburiae*	GCA_003289825	SAMN09435809	2016	France	Human	1065	IncHI1A, IncHI1B, IncFIB, Col440I, ColRNAI, Col440II
GEN000188	*E. hormaechei*	GCA_004146255	SAMN10847478	2017	France	Human	873	IncHI2, IncHI2A, RepA_1_pKPC-CAV1321
3849	*E. hormaechei*	GCA_012974405	SAMN14734641	2017	Italy	Human	382	IncHI2, IncHI2A, RepA_1_pKPC-CAV1321, IncN, Col440I, ColRNAI, Col440II
WP5-S18-CRE-02	*E. kobei*	GCA_014169295	SAMD00194526	2018	Japan	Environment	32	IncL/M, RepA_1_pKPC-CAV1321, Col440I, ColRNAI, Col440II
WP5-S18-ESBL-01	*E. asburiae*	GCA_014169315	SAMD00194528	2018	Japan	Environment	New	IncHI2, IncHI2A, RepA_1_pKPC-CAV1321, IncFIA, IncFIB, Col440I, ColRNAI
STN0717-73	*E. asburiae*	GCA_015138375	SAMD00195991	2018	Japan	Environment	484	IncHI2, IncHI2A, RepA_1_pKPC-CAV1321, IncFIB, IncFII, Col440I, ColRNAI, Col440II
KP0785	*E. cloacae*	GCA_015682695	SAMN06330157	2014	USA	Human	452	IncHI2, IncHI2A, RepA_1_pKPC-CAV1321, IncFIB, IncFII, IncFIA
MS14406	*E. cloacae*	GCA_015683115	SAMN14867396	2016	Australia	Human	167	IncHI2, RepA_1_pKPC-CAV1321, Col440I, ColRNAI, Col440II
NR1247	*E. cloacae*	GCA_015683195	SAMN06330201	2015	USA	Human	454	IncFIB, IncFII, IncN, Col440I, ColRNAI, Col440II
CQS45	*E. cloacae*	GCA_015683495	SAMN11230954	2016	Australia	Human	167	IncHI2, RepA_1_pKPC-CAV1321, Col440I, ColRNAI, Col440II
Ec_10_6	*E. cloacae*	GCA_015685455	SAMEA3886779	NA	NA	Human	167	IncHI2, IncHI2A, RepA_1_pKPC-CAV1321, Col440I, ColRNAI, Col440II
AZ_872	*E. cloacae*	GCA_015959265	SAMN07501554	2014	Nigeria	Human	836	IncFIB, IncHI1B, Col440I, ColRNAI, Col440II
176B9	*E. hormaechei*	GCA_016427675	SAMN17141544	2018	France	Human	873	IncHI2, IncHI2A, RepA_1_pKPC-CAV1321, IncFIB
189H4	*E. hormaechei*	GCA_016427965	SAMN17141574	2018	France	Human	873	IncHI2, IncHI2A, RepA_1_pKPC-CAV1321, IncN, Col440I, ColRNAI

192J3	*E. hormaechei*	GCA_016427975	SAMN17141576	2018	France	Human	873	IncHI2, IncHI2A, RepA_1_pKPC-CAV1321, IncFIB
184J3	*E. hormaechei*	GCA_016428155	SAMN17141567	2018	France	Human	873	IncHI2, IncHI2A, RepA_1_pKPC-CAV1321
183C10	*E. hormaechei*	GCA_016428465	SAMN17141562	2018	France	Human	873	IncHI2, IncHI2A, RepA_1_pKPC-CAV1321, IncFIB
182G8	*E. hormaechei*	GCA_016428495	SAMN17141561	2018	France	Human	873	IncHI2, IncHI2A, RepA_1_pKPC-CAV1321, IncFIB
1800	*E. hormaechei*	GCA_016428505	SAMN17141557	2018	France	Human	873	IncHI2, IncHI2A, RepA_1_pKPC-CAV1321, IncFIB
118G6	*E. hormaechei*	GCA_016632625	SAMN17137699	2016	France	Human	873	IncHI2, IncHI2A, RepA_1_pKPC-CAV1321, IncFIB
159B2	*E. hormaechei*	GCA_016632955	SAMN17137982	2017	France	Human	873	IncHI2, IncHI2A, RepA_1_pKPC-CAV1321, IncFIB, IncN, Col440I, ColRNAI, Col440II
168H9	*E. hormaechei*	GCA_016633045	SAMN17137990	2018	France	Human	873	IncHI2, IncHI2A, RepA_1_pKPC-CAV1321, IncFIB
136J9	*E. hormaechei*	GCA_016633055	SAMN17137947	2017	France	Human	873	IncHI2, IncHI2A, RepA_1_pKPC-CAV1321, IncFIB
146C8	*E. hormaechei*	GCA_016633425	SAMN17137963	2017	France	Human	873	IncHI2, IncHI2A, RepA_1_pKPC-CAV1321, IncFIB, IncN, Col440I, ColRNAI
145H10	*E. hormaechei*	GCA_016633475	SAMN17137960	2017	France	Human	873	IncHI2, IncHI2A, RepA_1_pKPC-CAV1321, IncFIB, IncN, Col440I, ColRNAI, Col440II
134B5	*E. hormaechei*	GCA_016633505	SAMN17137942	2017	France	Human	873	IncHI2, IncHI2A, RepA_1_pKPC-CAV1321, IncFIB
141I4	*E. hormaechei*	GCA_016633515	SAMN17137951	2017	France	Human	873	IncHI2, IncHI2A, RepA_1_pKPC-CAV1321, IncFIB, IncN, Col440I, ColRNAI, Col440II
133F1	*E. hormaechei*	GCA_016633605	SAMN17137941	2017	France	Human	873	IncHI2, IncHI2A, RepA_1_pKPC-CAV1321, IncFIB
128d3	*E. hormaechei*	GCA_016633615	SAMN17137713	2017	France	Human	873	IncHI2, IncHI2A, RepA_1_pKPC-CAV1321, IncFIB
117C1	*E. hormaechei*	GCA_016633995	SAMN17137696	2016	France	Human	873	IncHI2, IncHI2A, RepA_1_pKPC-CAV1321, IncFIB
80F6	*E. hormaechei*	GCA_016634305	SAMN17137673	2015	France	Human	873	IncHI2, IncHI2A, RepA_1_pKPC-CAV1321, IncFIB
82G3	*E. hormaechei*	GCA_016634365	SAMN17137675	2015	France	Human	873	IncHI2, IncHI2A, RepA_1_pKPC-CAV1321, IncFIB
BH3741	*E. cloacae*	GCA_020673785	SAMN20378227	2015	Nigeria	Human	836	IncHI2, IncHI2A, RepA_1_pKPC-CAV1321, Col440I, ColRNAI
03-010-0541	*E. cloacae*	GCA_020673815	SAMN20378232	2015	Nigeria	Human	836	IncHI2, IncHI2A, RepA_1_pKPC-CAV1321, Col440I, ColRNAI
PO1353	*E. cloacae*	GCA_020675995	SAMN20378228	2010	Nigeria	Human	836	IncHI2, IncHI2A, RepA_1_pKPC-CAV1321, Col440I, ColRNAI
MDR0253	*E. cloacae*	GCA_900075545	SAMEA2273337	2006	UK	Human	519	IncHI2, IncHI2A, RepA_1_pKPC-CAV1321, Col440I, ColRNAI, Col440II
e478	*E. hormaechei*	GCA_900077205	SAMEA2298375	2014	UK	Human	New	IncHI2, IncHI2A, RepA_1_pKPC-CAV1321, Col440I, ColRNAI
MDR0108	*E. asburiae*	GCA_900077545	SAMEA2273206	2003	UK	Human	610	IncHI2, IncHI2A, RepA_1_pKPC-CAV1321, Col440I, ColRNAI, IncFIA, IncFII
MDR0163	*E. hormaechei*	GCA_900078025	SAMEA2273264	2004	UK	Human	267	IncHI2, IncHI2A, RepA_1_pKPC-CAV1321, IncFIB, Col440I, ColRNAI, IncHI1B
MGYG-HGUT-	*E. asburiae*	GCA_902387755	SAMEA5852007	2019	NA	Human	1065	IncHI1A, IncHI1B, IncFIB, Col440I, ColRNAI, Col440II

02502								
KR2727	*E. hormaechei*	GCA_902713455	SAMEA5983686	2011	China	Human	93	IncHI2, IncHI2A, RepA_1_pKPC-CAV1321, Col440I, ColRNAI, IncR, IncFII
KR2728	*E. hormaechei*	GCA_902713475	SAMEA5983687	2011	China	Human	93	IncHI2, IncHI2A, RepA_1_pKPC-CAV1321, Col440I, ColRNAI, IncR, IncFII, IncX4
AI2613	*E. hormaechei*	GCA_903993075	SAMEA7082316	2017	Spain	Human	93	IncHI2, IncHI2A, RepA_1_pKPC-CAV1321, IncR, IncFII, Col440I, ColRNAI
AN2360	*E. hormaechei*	GCA_903993235	SAMEA7082339	2017	Spain	Human	93	IncHI2, IncHI2A, RepA_1_pKPC-CAV1321, IncR, IncFII, Col440I, ColRNAI
AI2705	*E. asburiae*	GCA_905219205	SAMEA7853274	2016	Spain	Human	515	IncFIB, IncFII, IncP, Col440I, ColRNAI, Col440II
AI2719	*E. asburiae*	GCA_905219325	SAMEA7853285	2016	Spain	Human	515	IncFIB, IncFII, IncFIA, Col440I, ColRNAI
AI2688	*E. hormaechei*	GCA_905231985	SAMEA7962766	2018	Spain	Human	78	IncHI2, IncHI2A, RepA_1_pKPC-CAV1321, IncFIB, IncFII, IncFIA, IncL/M, Col440I, ColRNAI
AI2689	*E. hormaechei*	GCA_905232005	SAMEA7962767	2018	Spain	Human	171	IncHI2, IncHI2A, RepA_1_pKPC-CAV1321, IncL/M, ColRNAI, Col440I
AI2656	*E. hormaechei*	GCA_905232475	SAMEA7962737	2018	Spain	Human	133	IncHI2, IncHI2A, RepA_1_pKPC-CAV1321, Col440I, ColRNAI, Col440II
AI2657	*E. hormaechei*	GCA_905232485	SAMEA7962738	2018	Spain	Human	133	IncHI2, IncHI2A, RepA_1_pKPC-CAV1321, Col440I, ColRNAI
AI2658	*E. hormaechei*	GCA_905232495	SAMEA7962739	2018	Spain	Human	133	IncHI2, IncHI2A, RepA_1_pKPC-CAV1321, Col440I, ColRNAI, Col440II
AI2662	*E. hormaechei*	GCA_905232535	SAMEA7962743	2018	Spain	Human	171	IncHI2, IncHI2A, RepA_1_pKPC-CAV1321, IncL/M, Col440I, ColRNAI
AI2760	*E. hormaechei*	GCA_905232815	SAMEA7962787	2018	Spain	Human	182	IncFIB, IncFII, IncL/M, Col440I, ColRNAI
AI2797	*E. asburiae*	GCA_905233045	SAMEA7962813	2018	Spain	Human	24	IncFIB, IncFII, IncHI2, IncHI2A, RepA_1_pKPC-CAV1321, Col440I, ColRNAI, Col440II
AI2798	*E. hormaechei*	GCA_905233055	SAMEA7962814	2018	Spain	Human	78	IncHI2, IncHI2A, RepA_1_pKPC-CAV1321, IncFIB
AI2799	*E. hormaechei*	GCA_905233065	SAMEA7962815	2018	Spain	Human	1015	IncHI2, IncHI2A, RepA_1_pKPC-CAV1321, IncFIB, IncL/M
AI2802	*E. asburiae*	GCA_905233075	SAMEA7962818	2018	Spain	Human	24	IncFIB, IncFII, IncHI2, IncHI2A, RepA_1_pKPC-CAV1321, Col440I, ColRNAI, Col440II
AI2747	*E. hormaechei*	GCA_905329565	SAMEA8065826	2018	Spain	Human	182	IncFIB, IncFII, IncHI2, IncHI2A, RepA_1_pKPC-CAV1321
AI2804	*E. hormaechei*	GCA_905329655	SAMEA8065845	2018	Spain	Human	78	IncFIB, IncFII , IncHI2, IncHI2A, RepA_1_pKPC-CAV1321
AI2936	*E. hormaechei*	GCA_905330775	SAMEA8065771	2018	Spain	Human	78	IncHI2, IncHI2A, RepA_1_pKPC-CAV1321, IncFIB, Col440I, ColRNAI, Col440II
AI2939	*E. hormaechei*	GCA_905330795	SAMEA8065774	2018	Spain	Human	133	IncHI2, IncHI2A, RepA_1_pKPC-CAV1321, Col440I, ColRNAI, Col440II
AI2984	*E. asburiae*	GCA_905331215	SAMEA8065815	2016	Spain	Human	515	IncFIB, IncFII, Col440I, ColRNAI, Col440II
AI3013	*E. hormaechei*	GCA_905331365	SAMEA8065846	2018	Spain	Human	764	IncHI2, IncHI2A, RepA_1_pKPC-CAV1321, IncFIB, IncFII, IncFIA, Col440I, ColRNAI, Col440II

51118	*E. hormaechei*	GCA_021285885	2014	China	Human	97	IncHI2, IncHI2A, RepA_1_pKPC-CAV1321, Col440I, ColRNAI
52272	*E. hormaechei*	GCA_021285525	2014	China	Human	116	IncHI2, IncHI2A, RepA_1_pKPC-CAV1321
52319	*E. hormaechei*	GCA_021285505	2014	China	Human	116	IncHI2, IncHI2A, RepA_1_pKPC-CAV1321
52744	*E. kobei*	GCA_021285945	2014	China	Human	1034	IncHI2, IncHI2A, RepA_1_pKPC-CAV1321
53287	*E. hormaechei*	GCA_021285905	2015	China	Human	600	IncFIB, IncFII, IncX3, Col440I, ColRNAI, Col440II
54401	*E. hormaechei*	GCA_021285655	2015	China	Human	50	IncHI2, IncHI2A, RepA_1_pKPC-CAV1321, Col440I, ColRNAI, Col440II
54570	*E. hormaechei*	GCA_021285645	2015	China	Human	50	IncHI2, IncHI2A, RepA_1_pKPC-CAV1321, Col440I, ColRNAI, Col440II
58918	*E. kobei*	GCA_021285445	2016	China	Human	56	IncX3, IncFIB, IncFII, Col440I, ColRNAI, Col440II
58941	*E. hormaechei*	GCA_021285385	2016	China	Human	97	IncHI2, IncHI2A, RepA_1_pKPC-CAV1321, IncFIA, IncFII
61363	*E. hormaechei*	GCA_021285405	2017	China	Human	90	IncHI2, IncHI2A, RepA_1_pKPC-CAV1321, IncN
60403	*E. hormaechei*		2010	China	Human	93	IncFIB, IncHI2, IncHI2A, RepA_1_pKPC-CAV1321

* NA, not available.

Table S2. Susceptibility profiles and MICs for 11 clinical isolates and their transconjugants.

Isolate/ transconjugant*	AMC	TZP	CTX	CAZ	CPO	ATM	IMP	MEM	GEN	AMK	LVX	CIP	SXT	FLR	TCY	FOS	CHL	COL	TGC	Conjugation temperature (°C)
51118	64	2	8	128	4	>128	0.25	0.03	>128	2	4	4	>152	32	>128	8	>64	0.5	0.5	
TC-51118	64	2	8	64	2	>128	0.25	0.03	>128	2	4	4	>152	16	>128	8	>64	0.5	0.5	26
52272	128	2	16	64	8	>128	0.25	0.06	16	16	0.5	0.5	>152	16	64	4	>64	1	0.25	
TC-52272	16	1	8	32	4	32	0.25	0.03	8	8	0.25	0.5	>152	16	32	0.5	>64	1	≤0.125	26
52319	64	16	32	128	16	>128	0.25	0.06	32	16	4	2	>152	>64	>128	4	32	2	2	
TC-52319	16	4	8	16	2	32	0.25	0.03	8	8	0.125	0.25	>152	8	32	0.5	8	0.5	≤0.125	37
52744	128	4	32	128	8	>128	0.25	0.06	8	2	0.5	1	>152	16	4	16	>64	64	0.25	
TC-52744	64	2	32	64	8	128	0.25	0.06	8	2	0.5	0.5	>152	16	4	8	>64	16	0.25	37
54401	128	2	16	64	8	>128	0.5	0.03	8	2	0.25	0.5	>152	16	4	16	>64	1	≤0.125	
TC-54401	16	2	8	64	4	128	0.5	0.03	8	2	0.25	0.5	>152	16	4	8	>64	0.5	≤0.125	26
54570	64	2	8	128	2	>128	0.25	≤0.015	>128	2	0.25	0.5	>152	16	>128	64	8	1	0.25	
TC-54570	64	2	8	128	0.5	64	0.25	0.03	>128	2	0.25	0.5	>152	8	128	0.5	8	0.5	≤0.125	37
58918	64	>128	>128	>128	64	>128	2	4	>128	2	0.25	1	≤2.375	16	>128	>256	>64	128	0.25	NA
58941	64	2	32	64	16	128	0.25	0.03	>128	16	16	16	>152	16	>128	16	16	2	≤0.125	
TC-58941	64	1	4	32	0.5	128	0.25	0.03	>128	16	0.25	0.5	>152	8	>128	0.5	8	1	≤0.125	26
61363	128	128	>128	>128	64	4	8	4	8	2	1	1	>152	16	4	16	16	1	0.5	
TC-61363	128	128	>128	>128	64	4	8	4	4	2	0.5	1	>152	8	2	2	8	1	≤0.125	37
60403	128	>128	>128	>128	64	128	0.5	0.5	>128	>128	16	64	>152	32	>128	>256	>64	1	0.5	NA
53287	128	>128	>128	>128	>128	4	8	2	8	2	0.06	0.06	≤2.375	16	4	8	16	1	0.25	NA
J53	16	1	0.06	0.25	0.125	0.125	0.25	0.03	1	1	0.015	0.03	≤2.375	8	2	0.5	8	1	≤0.125	

*TC, transconjugants; MIC values (mg/ml); AMC: Amoxicillin/clavulanic acid; TZP: Piperacillin/tazobactam; CTX: Cefotaxime; CAZ: Ceftazidime; CPO: Cefpirome; ATM: Aztreonam; IMP: Imipenem; MEM: Meropenem; GEN: Gentamicin; AMK: Amikacin; LVX: Levofloxacin; CIP: Ciprofloxacin; SXT: Trimethoprim/sulfamethoxazole; FLR: Florfenicol; TCY: Tetracycline; FOS: Fosfomycin; CHL: Chloramphenicol; COL: Colistin; TGC: Tigecycline; NA, not applicable.

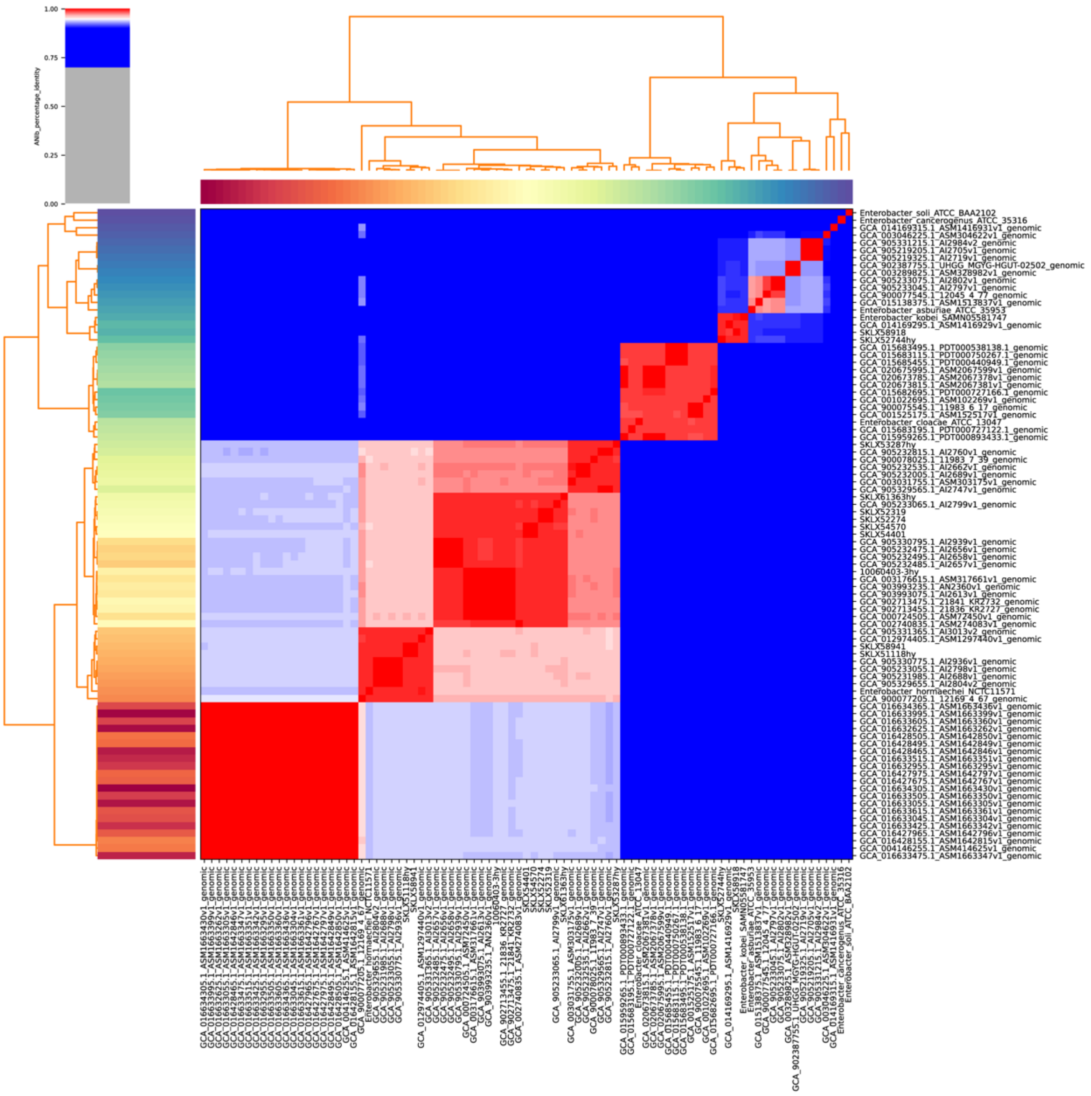

Figure S1. ANIb analysis of MCR-9-producing ECC isolates.

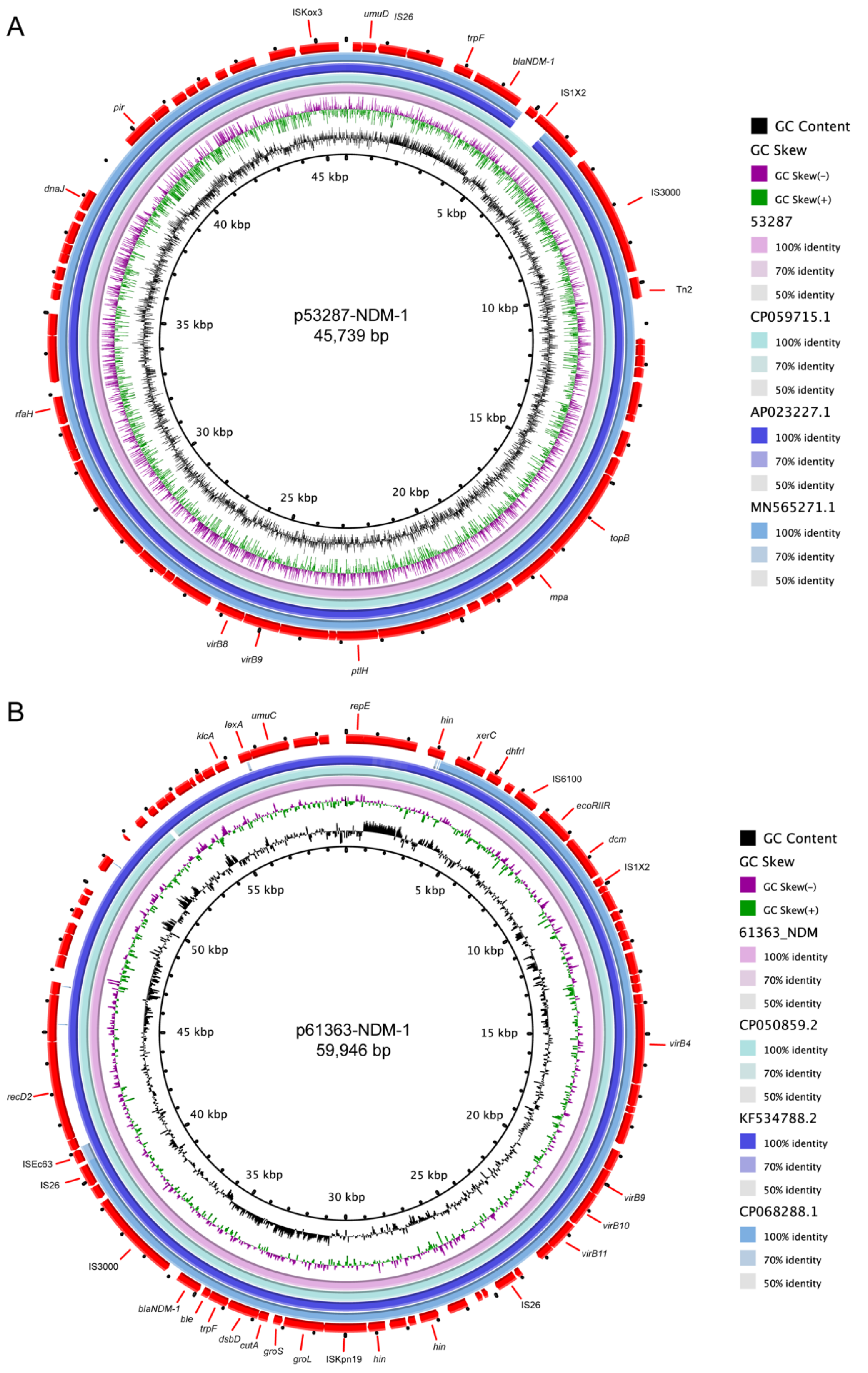

Figure S2. Circular representation of the studied *bla*$_{NDM-1}$-carrying plasmids. A) Sequence comparison of the IncX3 plasmid p53287-NDM-1carried by the bacteremia associated with *E. hormaechei* strain 53287. B) Sequence comparison of the *bla*$_{NDM-1}$-

carrying IncN plasmid p61363-NDM-1. GC content and GC Skew were represented on the inner map's distance scale (in kbp). Each plasmid was compared to its most closely-related plasmid. The red arc around the map indicated ORFs. Certain important genes were also indicated on the ring.
